# Modulation of Cellular Circadian Rhythms by Secondary Metabolites of Lichens

**DOI:** 10.3389/fncel.2022.907308

**Published:** 2022-06-23

**Authors:** Soumi Srimani, Cosima Xenia Schmidt, Maria Pilar Gómez-Serranillos, Henrik Oster, Pradeep K. Divakar

**Affiliations:** ^1^Institute of Neurobiology, Center of Brain, Behavior & Metabolism (CBBM), University of Lübeck, Lübeck, Germany; ^2^Department of Pharmacology, Pharmacognosy and Botany, Faculty of Pharmacy, Complutense University of Madrid, Madrid, Spain

**Keywords:** evernic acid, usnic acid, circadian clocks, amplitude, dampening, *in vitro* models

## Abstract

**Background:**

Most mammalian cells harbor molecular circadian clocks that synchronize physiological functions with the 24-h day-night cycle. Disruption of circadian rhythms, through genetic or environmental changes, promotes the development of disorders like obesity, cardiovascular diseases, and cancer. At the cellular level, circadian, mitotic, and redox cycles are functionally coupled. Evernic (EA) and usnic acid (UA), two lichen secondary metabolites, show various pharmacological activities including anti-oxidative, anti-inflammatory, and neuroprotective action. All these effects have likewise been associated with a functional circadian clock.

**Hypothesis/Purpose:**

To test, if the lichen compounds EA and UA modulate circadian clock function at the cellular level.

**Methods:**

We used three different cell lines and two circadian luminescence reporter systems for evaluating dose- and time-dependent effects of EA/UA treatment on cellular clock regulation at high temporal resolution. Output parameters studied were circadian luminescence rhythm period, amplitude, phase, and dampening rate.

**Results:**

Both compounds had marked effects on clock rhythm amplitudes and dampening independent of cell type, with UA generally showing a higher efficiency than EA. Only in fibroblast cells, significant effects on clock period were observed for UA treated cells showing shorter and EA treated cells showing longer period lengths. Transient treatment of mouse embryonic fibroblasts at different phases had only minor clock resetting effects for both compounds.

**Conclusion:**

Secondary metabolites of lichen alter cellular circadian clocks through amplitude reduction and increased rhythm dampening.

## Introduction

The circadian clock system helps to maintain the adaptation of physiological and psychological functions to the changing environmental conditions induced by the Earth’s rotation around its axis (Neumann et al., 2019). Studies reported that most cells and tissues of our body harbor molecular clocks which are in synchronization with day-night rhythms and coordinated by the suprachiasmatic nucleus (SCN) of the hypothalamus (Stephan and Zucker, 1972; Balsalobre et al., 1998; Yamazaki et al., 2000). At the cellular level, clock genes *Clock* (*Circadian Locomotor Output Cycles Kaput*), *Bmal1* (*Brain and Muscle ARNT-Like 1*), *Per1* (*Period 1*), *Per2* (*Period 2*), *Cry1* (*Cryptochrome 1*), *Cry*2 (*Cryptochrome 2*), and *Rev-Erbα* regulate these circadian rhythms (Hastings and Herzog, 2004). Interlocked transcriptional-translational feedback loops control these clock genes (Reppert and Weaver, 2002; Bell-Pedersen et al., 2005; Rosbash et al., 2007). Factors like sedentary lifestyle, prolonged stress or consumption of processed foods or high-sugar or high-fat diets alter circadian rhythms of cells and tissues and promote the development of a range of cardio-metabolic disorders and cancers (Tahara et al., 2015; Panda, 2016; Husse et al., 2017; Kiehn et al., 2017; Neumann et al., 2019). Many of these factors also result in the cellular accumulation of pro-oxidants and induce oxidative stress (OS), an imbalance between intracellular production of reactive oxygen species (ROS) and antioxidant defense mechanism (Charradi et al., 2013; Sharifi-Rad et al., 2020). OS activates a series of transcription factors including NF-κβ (nuclear factor kappa-light-chain-enhancer of activated B cells), AP-1 (Activator protein 1), p53 (Tumor Protein p53), HIF-1α (Hypoxia-inducible factor 1-alpha), PPAR-γ (Peroxisome proliferator-activated receptor gamma), β-catenin/Wnt, and *Nrf2* (nuclear factor erythroid 2-related factor 2; Reuter et al., 2010). Activation of these transcription factors results in the expression of more than 500 different genes encoding for growth factors, inflammatory cytokines, chemokines, cell cycle regulators, and anti-inflammatory molecules, and hence OS results in cytotoxic effects in many mammalian organs and tissues (Behl et al., 1994; Uttara et al., 2009; Reuter et al., 2010). Increased ROS generation and decreased antioxidative enzyme activity (like CAT, SOD, GPx, and GST) have been reported in animals deficient in clock proteins, supporting the evidence that the activity and expression of these genes in different brain regions follow the diurnal rhythm and thus they are under the control of the endogenous circadian system (Pablos et al., 1998; Baydas et al., 2002; Kondratov et al., 2006; Krishnan et al., 2008; Yuan et al., 2017). Hence, along with altered circadian rhythms, prolonged OS also leads to sustained inflammation and chronic diseases including obesity, cancer, diabetes, cardiovascular, neurodegenerative, and pulmonary disorders (Lu and Zee, 2006; Perwez Hussain and Harris, 2007; Reuter et al., 2010; Tahara et al., 2015; Panda, 2016; Sharifi-Rad et al., 2020).

Lichens are symbiotic organisms between a fungal (the mycobiont) and an algal and/or cyanobacterial (the photobiont) partner (Calcott et al., 2018). In addition to the predominant myco- and photobionts, additional fungi, and non-photosynthetic bacteria are often associated with a lichen thallus (Grube et al., 2015; Spribille et al., 2016; Smith et al., 2020). Primary metabolites (such as simple sugars, ribitol, or glucose) produced by the photobiont partner of lichens are used for the mycobiont’s nutrition (Brodo et al., 2001; Calcott et al., 2018; Spribille et al., 2022). Lichens are well known to produce a large number of secondary metabolites with almost 1,000 known substances, the large majority of which are exclusively found in lichen forming fungi (Gómez-Serranillos et al., 2014; Calcott et al., 2018). These are deposited extracellularly, mainly in the medullary layer of lichen thallus or in the cortical layer. Phenolic compounds that originate from polyketide pathways, such as depsides, depsidones, and usnic acids are found almost exclusively in lichens. Atranorin and usnic acid are the most common and abundant cortical substances in lichens. They help in protecting the organism from UV radiation, environmental toxicity, pathogens, herbivores, and from other physical hazards (Ranković and Kosanić, 2015). Evernic acid (EA; molecular weight: 332.308 g), a depside, is produced in the medullary layer mainly by a common lichen species, *Evernia prunastri*, usually found in oak trees (Ter Heide et al., 1975; Joulain and Tabacchi, 2009). Other than *Evernia prunastri*, lichen genera such as *Ramalina* and *Hypogymnia* also produce EA (Olivier-Jimenez et al., 2019). Usnic acid (UA; molecular weight: 344.315 g), a dibenzofuran derivative, occurs in two enantiomers and is most commonly found in the cortex of *Usnea* species (Shukla et al., 2010). Other than in *Usnea*, it is also found in several lichen genera as *Cladonia, Lecanora, Ramalina, Xanthoparmelia, Flavoparmelia* and *Alectoria* (Cocchietto et al., 2002).

The production of primary metabolites by the photobionts is highly regulated by external cues like light, temperature, moisture, and gaseous concentrations in the atmosphere (Kallio and Heinonen, 1971; Kershaw and Smith, 1978; Okada et al., 1978; Korhonen and Kallio, 1987; Carré and Edmunds, 1993; Ott and Schieleit, 1994; Mittag, 2001; Segovia et al., 2003; Dodd et al., 2005; Eymann et al., 2017; Cano-Ramirez et al., 2018). *kaiA*, *kaiB*, and *kaiC* are the core clock genes in cyanobacteria as the circadian rhythms of it have been studied vigorously (Kondo and Ishiura, 2000; Dvornyk et al., 2003). Whereas the circadian clock genes have not been largely studied in fungi, this has only been well documented in a model organism like *Neurospora crassa* (Collett et al., 2002; Froehlich et al., 2002; Dunlap and Loros, 2005, 2006). Highly conserved circadian clock homologs are present in most plants and algae, while white collar-1 (WC-1), the circadian core clock component in fungi shows greater similarity in the sequences with the animal core clock component BMAL1/CLOCK (Tauber et al., 2004; Linde et al., 2017; Brody, 2019). More recently, the presence of core circadian clock genes frequency (*frq*), *wc-1*, white collar-2 (*wc-2*), and Frequency Interacting RNA Helicase (*frh*) have been reported in a lichenized fungus (Valim et al., 2022). They have also reported that the *frq* gene was activated in a light-dependent manner, similar to *Neurospora crassa*. Though it remains unclear whether lichens as a holobiont have any circadian rhythms, and thus the biosynthesis of the secondary metabolites is probably controlled by the circadian rhythms of the lichens.

Lichenized secondary metabolites impart a wide range of pharmacological activities like antioxidant, neuroprotective, cytotoxic, antimicrobial, anti-inflammatory, analgesic, and enzyme inhibitory action (Gómez-Serranillos et al., 2014; Fernández-Moriano et al., 2015a; Ranković and Kosanić, 2015; Yamamoto et al., 2015; Solárová et al., 2020). Anti-proliferative and anti-tumoral effect of UA (10 μg/ml and 50 μg/ml concentrations) on human lung cancer tumoral cells by stimulating APOPT1, CYCS, APAF1, CASP3, and CASP9 genes expression, has also been reported (Çoban et al., 2017). Fernández-Moriano et al. (2017) reported the neuroprotective potential of UA and EA with strong radical scavenging properties (ORAC and DPPH tests) and improved cellular redox status (by inhibiting H_2_O_2_ induced intracellular ROS overproduction, by restoring GSH/GSSG ratio and by lowering lipid peroxidation level through MDA) for the first time on 2017 in two models of central nervous system-like cells (U373-MG and SH-SY5Y cell lines). They have also reported that EA pre-treatment also improved the enzymatic and non-enzymatic antioxidant defense through the activation of the Nrf2 signaling pathway. UA exerts significant but more moderate effects (Fernández-Moriano et al., 2017). Further, they could be used in the therapy of oxidative stress–related diseases (Sahin et al., 2019).

Oxidative stress, mitochondrial impairment, neuroinflammation, and impaired protein degradation are the most important pathophysiologies behind the onset of neurodegenerative diseases like Parkinson’s disease, Alzheimer’s disease, etc. (Mandel et al., 2003). The brain has higher uptake of glucose and oxygen, which causes neurodegeneration *via* OS-related dysregulation of Nrf2-ARE defense system (Cui et al., 2016). *Nrf2* is activated in response to OS which also regulates the gene expression of antioxidant enzymes, GSH-related genes, and antioxidant proteins *via* the antioxidant responsive element (ARE) in a cell-type dependent manner (Itoh et al., 1997; Ishii et al., 2000; Mann and Forman, 2015). Previous studies reported that astrocytes play a major role in GSH synthesis and thus protect neurons from oxidative stress induced degeneration (Sagara et al., 1993; Fernandez-Checa et al., 1997; Ishii and Mann, 2014). Moreover, circadian rhythms of antioxidative gene expression were altered by 6-hydroxydopamine, a neurotoxin *via* downregulating the expression of *Bmal1*, *Per2*, and other clock genes in both the *in vitro* and *in vivo* PD animal models (Wang et al., 2018). As disruption of the circadian rhythm may be associated with the inadequate inactivation of *Nrf2* and dysregulation of astrocyte-neuron interactions, control of the circadian clock is particularly important for the normalization of brain functions and neuronal protections. Previous studies show that EA and UA are highly lipophilic in nature and, thus, cross lipid bilayers to reach the cytosol and mitochondria (Joseph et al., 2009; Shcherbakova et al., 2021). Hence, we can speculate that lichenized substances impart radical scavenging activities by altering the core clock gene expressions which also activates *Nrf2* and prevents neurodegenerations. However, until now it remains unclear whether these varieties of pharmacological activities imparted by lichenized secondary metabolites take place by altering the circadian clocks of the organism. Thus, in this present study, we aimed to find out whether two lichen metabolites, EA and UA, modulate circadian clock function at the cellular level.

## Materials and Methods

### Reagents

DMEM (1×) Glutamax-I, DPBS, penicillin-streptomycin (P/S), fetal bovine serum (FBS), 1× 0.05% trypsin-EDTA (T/E), B-27, HEPES (1 M), and sodium bicarbonate 7.5% solution were purchased from Gibco, Thermo Fisher Scientific (Waltham, USA). EA and D-luciferin were purchased from Cayman Chemical, Ann Arbor, USA, and PanReac AppliChem, Darmstadt, Germany, respectively. DMEM low-glucose powder, D-(+)-glucose, UA, dexamethasone (Dex), and dimethyl-sulfoxide (DMSO) were purchased from Sigma Aldrich, St. Louis, USA. Acridine Orange Propidium Iodide (AO-PI) was purchased from Logos Biosystems, Gyeonggi-do, South Korea.

### Cell Culture

Human bone osteosarcoma epithelial cells (U2OS), originally known as 2T cells, were derived from the bone tissue of a female teenager suffering from osteosarcoma. U2OS cells exhibit epithelial adherent morphology (Ponten and Saksela, 1967). An embryonic mouse hypothalamic cell line (N44) was isolated and immortalized from mouse embryonic (days 15–18 *post coitum*) hypothalamic primary cultures by retroviral transfer of SV40-T antigen (Cedarlane, Burlington, Canada). An embryonic mouse fibroblast (MEF) cell line was created and immortalized from PER2::LUCIFERASE reporter mice (Yoo et al., 2005) following standard protocols. N44-BL (Tsang et al., 2020), U2OS-BL (Maier et al., 2009), and MEF-P2L circadian reporter cells were authenticated by short tandem repeat (STR) profiling and tested negative for mycoplasma by PCR. Cells were maintained in DMEM with 4.5 g/L of D-glucose, supplemented with 10% (FBS) and 10,000 U P/S at 37°C with 5% CO_2_. Cells stably expressing *Bmal1:luc* reporter *via* lentiviral transduction were generated polyclonally by puromycin selection (Brown et al., 2005; Lin et al., 2019; Tsang et al., 2020).

### Bioluminescence Assay

**Figure 1:**
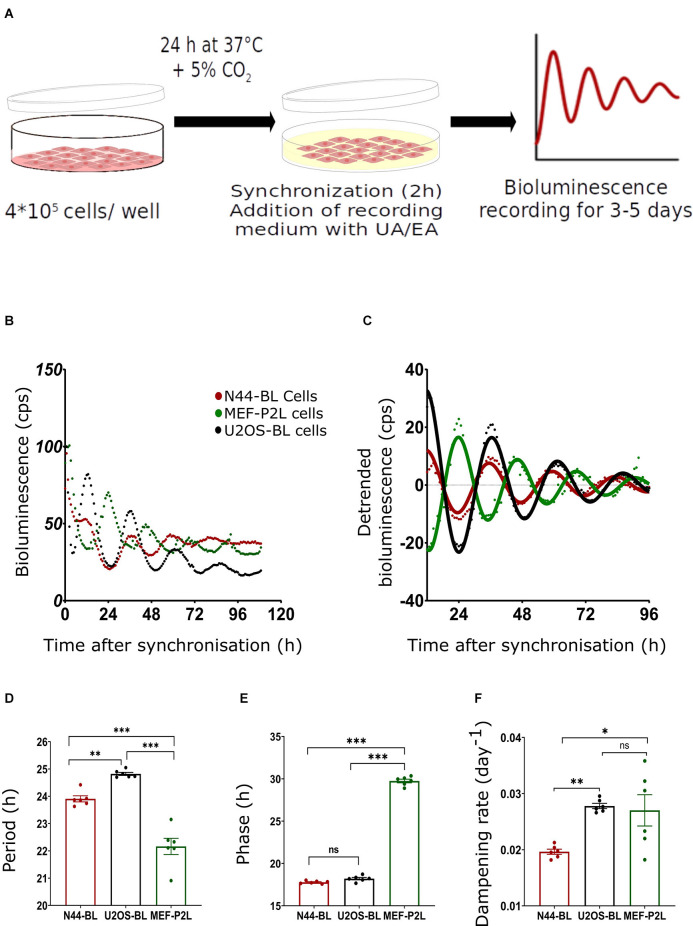
Characterization of circadian luminescence rhythms in N44, U2OS, and MEF cells. **(A)** Experimental setup; for more details, see text. **(B)** Representative raw and **(C)** representative normalized data and sine curve fittings of *Bmal1:luc* (BL) and *Per2:luc* (P2L) luminescence recordings of hypothalamus-derived mouse N44 (N44-BL), human bone osteosarcoma epithelial (U2OS-BL), and mouse embryonic fibroblast (MEF-P2L) cells after synchronization with dexamethasone (Dex). **(D–F)** Luminescence period lengths **(D)**, rhythm phases **(E)**, and dampening rates **(F)** of the same data set. Single measures are shown; error bars indicate means ± SEM (^*^p < 0.05, ^**^p < 0.01, ^***^p < 0.001; 1-way ANOVA with Dunnett’s *post-*test; *n* = 6). ns, non-significant.

The setup of the bioluminescence experiment is illustrated in Figure 1A. In brief, 2^*^10^5^ cells/ml of mouse hypothalamic (N44-BL), human osteosarcoma (U2OS-BL), and mouse embryonic fibroblast (MEF-P2L) cells stably expressing circadian clock reporter constructs (*Bmal1::luciferase—*BL or *Per2::luciferase—*P2L) were seeded in either 96-well plates (200 μl cell suspension/well) or 35-mm Petri dishes (2 ml cell suspension per dish) and grown to about 90% confluency (ca. 24 h at 37°C with 5% CO_2_). On the next day, cells were synchronized by adding 100 nM Dex for 2 h at 37°C with 5% CO_2_. To remove the phenol red from the DMEM, cells were washed with DPBS after the synchronization. After that, the medium was replaced with the same volume of recording medium (1 g/L DMEM low-glucose powder, 10 mM D-glucose, 3 mM 7.5% sodium bicarbonate, 10 mM HEPES, 1% 10,000 U penicillin/streptomycin, 2% B-27 supplement and 0.1 mM D-luciferin; Pilorz et al., 2020) in the dark. Plates were sealed with transparent film and luminescence was recorded for 3–5 days at 34°C using either Berthold TriStar LB 941 (Berthold Technologies, Wildbach, DE; N-44 and U2OS cells) or SpectraMax L 1-channel luminescence plate readers (Molecular Devices, San Jose, USA; MEF cells; Tsang et al., 2012). For the phase response experiment, cells were seeded in 35-mm Petri dishes. After 24 h incubation at 37°C with 5% CO_2_, cells were synchronized for 2 h with Dex followed by washing with DPBS and a change to the recording medium. Dishes were covered with glass coverslips and sealed with vacuum grease. Bioluminescence was measured at 32.5°C in LumiCycle 32 luminometer (Actimetrics, Evanston, USA; Tsang et al., 2012).

### Cell Treatment and Viability Assay

Usnic acid (UA; 2,6-Diacetyl-7,9-dihydroxy-8,9*b*-dimethyldibenzofuran-1,3(2*H*,9*bH*)-dione) and Evernic acid (EA; 2-hydroxy-4-[(2-hydroxy-4-methoxy-6-methylbenzoyl)oxy]-6-methyl-benzoic acid) were dissolved in dimethyl-sulfoxide (DMSO) followed by dilution in phosphate-buffered saline (PBS) to the desired concentrations (final concentration of DMSO lower than 0.1%). Cell viability assay was conducted after 24 h pre-treatment with different doses of secondary metabolites of lichens using AO-PI staining. Cells were seeded in 35-mm Petri dishes (2 ml cell suspension per dish) and grown to about 90% confluency (ca. 24 h at 37°C with 5% CO_2_). On the next day, cell suspension was discarded, followed by a wash with DPBS. Cells were pre-treated with different concentrations of secondary metabolites and control (PBS/DMSO), added with 2 ml of cell culture medium, and incubated for approximately 24 h at 37°C + 5% CO_2_. On the following day cells were washed with 1 ml of DPBS/ 35 mm dish. After that, 500 μl of T/E per 35 mm dish were added and incubated for approximately 2–3 min at 37°C + 5% CO_2_. 2 ml cell culture medium was added to every 35 mm Petri dish and the cell suspension was centrifuged at 200× g for 5 min followed by the removal of the supernatant and resuspension in 1 ml of cell culture medium per sample. Eighteen microliters of this cell suspension was mixed with 2 μl of AO-PI stain. Ten microliters of this mixture was transferred to a channel in the Ultra-low Fluorescence counting slide for the estimation of viable cells (in the percentage of the total cells) were recorded in the LUNA-FL Dual Fluorescence Cell Counter by Logos Biosystems, Gyeonggi-do, South Korea. 94.35%, 96.35%, 96.2%, 96.3%, and 71.8% (*n* = 2) N44-BL cells were viable upon 24 h pre-treatment with control along with 10 μg/ml, 25 μg/ml, 50 μg/ml, and 100 μg/ml of UA, respectively. Whereas, 98.9%, 97.8%, and 96.5% (*n* = 2) U2OS-BL cells were viable after 24 h pre-treatment with control along with 10 μg/ml and 50 μg/ml of UA, respectively. Moreover, significant differences in the rhythm parameters were absent in cells treated with <10 μg/ml and concentrations between 10 μg/ml to 50 μg/ml of EA or UA against control. We then proceeded with our final data analysis and representation with a lower, i.e., 10 μg/ml and a higher, i.e., 50 μg/ml concentrations treatment of the studied secondary metabolites of lichens as these concentrations were not lethal to the cells. For the dose-response experiments, cells were kept in a recording medium containing different concentrations (10 μg/ml and 50 μg/ml) of UE or EA. Bioluminescence was recorded for 3–5 days. Control cells were exposed to recording medium containing the same volume of PBS/DMSO. Same doses of UA, EA or Control were added to the cells after 5 days of recording for resynchronization and bioluminescence was recorded for another 2–3 days. For phase response experiments, bioluminescence recordings were started after the addition of recording medium to the cells. At the indicated time points, equal volumes of PBS/DMSO, UA, or EA were added individually to the designated dishes without wash-out.

### Data Transformation and Statistical Analysis

Circadian parameters of luminescence recordings were determined by individually adjusting for long-term trends in raw luminescence readouts by 24-h moving average baseline subtraction as described (Tsang et al., 2020). A damped sine wave $\left[y\left(t\right)=amplitude*e^{-dampening\;rate*t}*\sin\left(\frac{2\pi }{period}t+phase\;angle\right)\right]$ was fitted to baseline-adjusted data after testing for rhythmicity using the Cosinor algorithm (Nelson et al., 1979; Refinetti, 2004). Phase shifts were determined by comparing the fit peak times of substance and PBS/DMSO treated cells (Tsang et al., 2012). Amplitude effects were calculated after normalizing to starting values to account for differences in cell density or reporter signal. Treatment times for phase response profiles were determined *post hoc* using the intersection of the ascending cross-section of the sine fit with the x-axis as a reference and converted into degrees. Dampening rate constants of peak magnitudes were calculated from damped sine wave regressions using GraphPad Prism 9.1.2 software (GraphPad, La Jolla, USA). All statistical analyses were performed using GraphPad Prism. Two-tailed unpaired Student’s *t*-tests were used for pairwise comparisons. Multi-group analyses were performed by 1-way ANOVA and Dunnett’s *post-hoc* test. To compare changes in rhythm parameters in dose response experiments, 2-way ANOVAs with Tukey’s *post-hoc* test was used. A *p*-value of < 0.05 was used as cut-off for significance (Tsang et al., 2012, 2020).

## Results

### Quantitative Characterization of Circadian Luminescence Rhythms in Three Different Cell Lines

Figure 1B depicts the representative raw bioluminescence recordings of all three cell lines. Figure 1C represents the curve-fitted and detrended rhythm of the same data set. For the determination of circadian rhythm parameters (period, phase, and dampening rate) damped sine curves were fitted to the normalized data. The mean period lengths of MEF-P2L, N44-BL, and U2OS-BL cells were 22.2 ± 0.3 h, 23.9 ± 0.1 h, and 24.8 ± 0.06 h, respectively (Figure 1D). Significant differences in period length between all three cell lines were found. Phase shifts (Figure 1E) were determined by comparing the fit peak times in these cells. The mean phases of MEF-P2L, N44-BL, and U2OS-BL cells were 29.74 ± 0.21 h, 17.72 ± 0.07 h, and 18.34 ± 0.14 h, respectively. Dampening rate constants of peak magnitudes (Figure 1F) were calculated from damped sine wave regressions. Mean dampening rates of MEF-P2L, N44-BL, and U2OS-BL cells were 0.027 ± 0.003 h^−1^, 0.020 ± 0.001 h^−1^, and 0.028 ± 0.001 h^−1^, respectively. Significant phase differences were observed between MEF-P2L cells and the other two cell lines. Significant differences in dampening rates were observed between N44-BL cells and the other two cell lines. In summary, irrespective of the type of cell line and the luciferase reporter among untreated cells, significantly different period length and dampening rates were observed. As expected from the feedback nature of the circadian molecular clockwork (Figure 1E), phasing was significantly different between P2L and BL rhythms.

### Lichen Metabolite-Induced Dose- and Cell Type-Dependent Changes in Circadian Luminescence Rhythms

**Figure 2:**
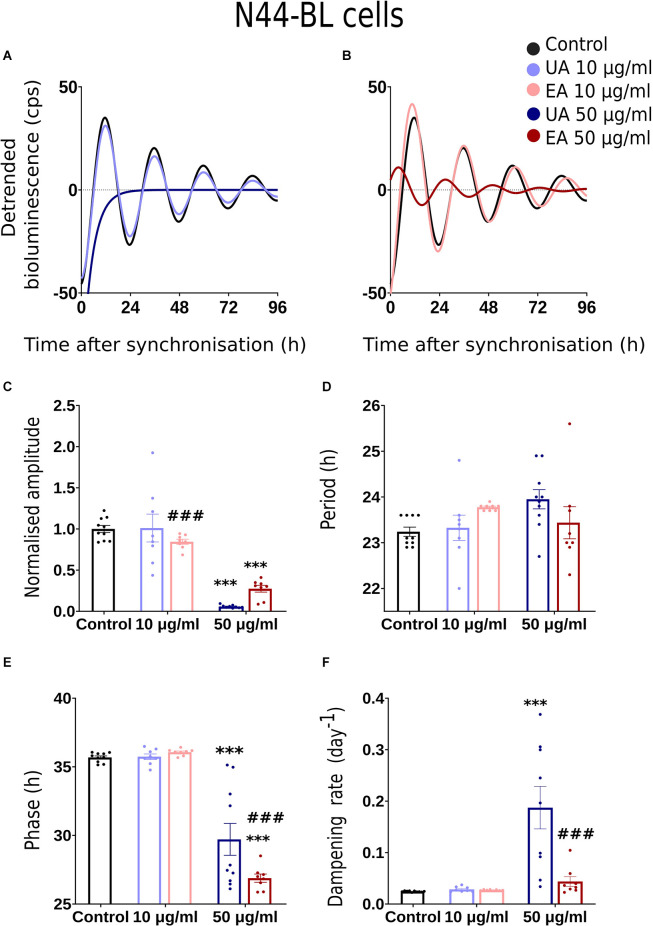
Effects of lichenized secondary metabolites on circadian luminescence rhythms of N44-BL cells. Representative curve fits of *Bmal1:luc* luminescence rhythms in N44 cells after treatment with different doses of usnic acid (**A**, UA/Blue) or evernic acid (**B**, EA/Red). Amplitude **(C)**, Period **(D)**, Phase **(E)**, and Dampening effects **(F)** on bioluminescence rhythms in N44 cells after treatment with EA (10 or 50 μg/ml; red) or UA (10 or 50 μg/ml; blue) vs. solvent control (PBS/ DMSO; black). Single measures are shown; error bars indicate means ± SEM (^***^p < 0.001; 2-way ANOVA with Tukey’s post-test). ^###p^ < 0.001 between UA 10 vs. EA 10 or UA 50 vs. EA 50.

**Figure 3:**
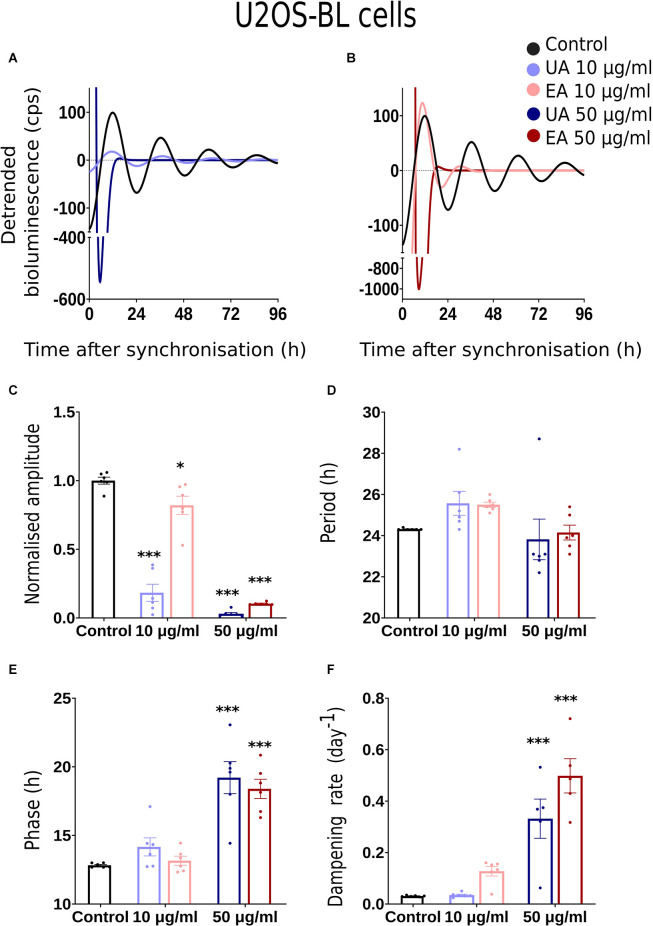
Effects of lichenized secondary metabolites on circadian luminescence rhythms of U2OS-BL cells. Representative curve fits of *Bmal1:luc* luminescence rhythms in U2OS cells after treatment with different doses of usnic acid (**A**, UA/Blue) or evernic acid (**B**, EA/Red). Amplitude **(C)**, Period **(D)**, Phase **(E)**, and Dampening effects **(F)** on bioluminescence rhythms in U2OS cells after treatment with EA (10 or 50 μg/ml; red) or UA (10 or 50 μg/ml; blue) vs. solvent control (PBS/ DMSO; black). Single measures are shown; error bars indicate means ± SEM (^*^p < 0.05, ^***^p < 0.001; 2-way ANOVA with Tukey’s post-test).

**Figure 4:**
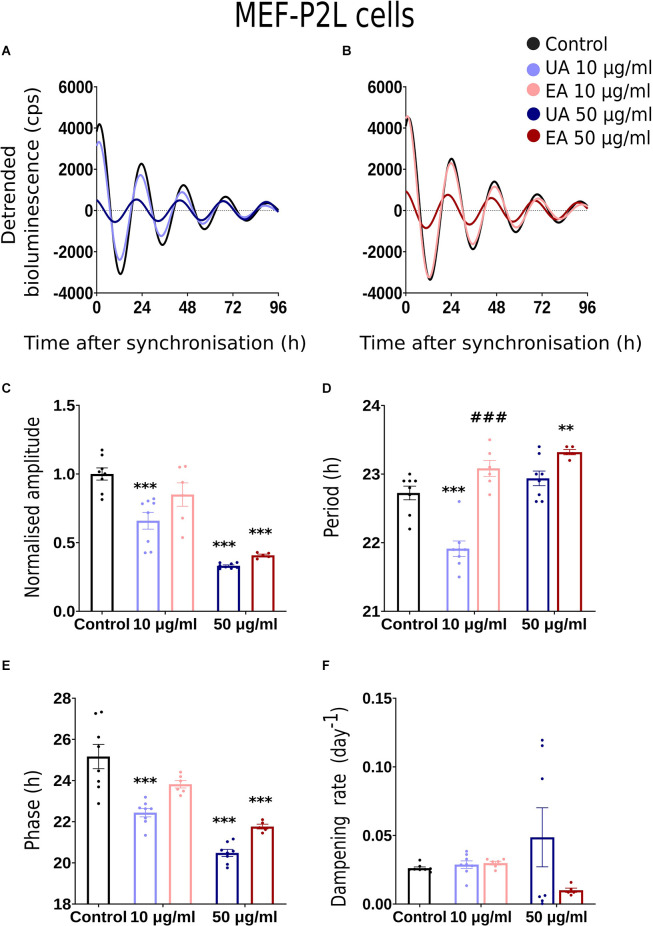
Effects of lichenized secondary metabolites on circadian luminescence rhythms of MEF-P2L cells. Representative curve fits of *Per2:luc* luminescence rhythms in MEF cells after treatment with different doses of usnic acid (**A**, UA/Blue) or evernic acid (**B**, EA/Red). Amplitude **(C)**, Period **(D)**, Phase **(E)**, and Dampening effects **(F)** on bioluminescence rhythms in MEF cells after treatment with EA (10 or 50 μg/ml; red) or UA (10 or 50 μg/ml; blue) vs. solvent control (PBS/ DMSO; black). Single measures are shown; error bars indicate means ± SEM (^**^p < 0.01, ^***^p < 0.001; 2-way ANOVA with Tukey’s post-test). ^###p^ < 0.001 between UA 10 vs. EA 10 or UA 50 vs. EA 50.

We conducted dose-response bioluminescence experiments in all the three cell lines to compare the extents to which different concentrations of UA or EA affect cellular P2L and BL rhythms. Supplementary Figures S1A,C depict the representative raw bioluminescence recording in N44-BL cells treated with different doses of UA (2.5 μg/ml, 5 μg/ml, 10 μg/ml and 50 μg/ml; Supplementary Figure S1A) and EA (5 μg/ml, 10 μg/ml, 25 μg/ml, 50 μg/ml and 100 μg/ml; Supplementary Figure S1C). Curve-fitted and detrended rhythms of the same data sets are presented under Supplementary Figure S1B (UA) and Supplementary Figure S1D (EA). For resynchronization, after 5 days of recording, the same doses of UA, EA, or Control were added to the cells with the pre-treatment and bioluminescence was recorded for another 2–3 days. In our exploratory analyses, significant resynchronization effect by the lichenized substances was not seen in any of the experiment (data are not shown here). Supplementary Figures S1A,B shows the representative raw (Supplementary Figure S1A) and curve-fitted detrended (Supplementary Figure S1B) bioluminescence *Bmal1:luc* rhythm of the N44 cells treated with different doses of UA against PBS/DMSO control. A significant difference in the rhythm parameters was absent in cells treated with <10 μg/ml and concentrations between 10 μg/ml to 50 μg/ml of EA or UA against control (data are not shown here). Whereas, cells became arrhythmic upon treatment with 100 μg/ml of EA (Supplementary Figures S1C,D). We speculated that the dampening in the rhythm at 50 μg/ml and arrhythmicity upon 100 μg/ml of UA or EA could be due to the cell death in such higher concentrations. We then conducted a cell viability assay using AO-PI stain in the N44 and U2OS cells after 24 h pre-treatment with different concentrations of UA against PBS/ DMSO control. 94.35%, 96.35%, 96.2%, 96.3%, and 71.8% (*n* = 2) N44-BL cells were viable upon 24 h pre-treatment with control along with 10 μg/ml, 25 μg/ml, 50 μg/ml and 100 μg/ml of UA, respectively. Whereas, 98.9%, 97.8%, and 96.5% (*n* = 2) U2OS-BL cells were viable after 24 h pre-treatment with control along with 10 μg/ml and 50 μg/ml of UA, respectively. We then proceeded with our final data analysis and representation with a lower, i.e., 10 μg/ml and a higher, i.e., 50 μg/ml concentrations treatment of the studied secondary metabolites of lichens as these concentrations were not lethal to the cells. Figures 2A, 3A, 4A show representative BL and P2L rhythms of the respective N44-BL (Figure 2A), U2OS-BL (Figure 3A), and MEF-P2L cells (Figure 4A) treated with 10 and 50 μg/ml of UA against solvent control. On the other hand, Figures 2B, 3B, 4B depict representative BL and P2L curve-fitted normalized luminescence rhythms upon treatment with 10 and 50 μg/ml of EA against solvent control in N44-BL (Figure 2B), U2OS-BL (Figure 3B), and MEF-P2L cells (Figure 4B), respectively. Irrespective of reporter and cell type, 50 μg/ml of UA and EA yielded robust dampening of the luminescence rhythm. UA at 50 μg/ml dampened the BL rhythm more strongly than that of P2L. In general, BL rhythms in U2OS cells showed greater sensitivity towards any concentrations of both secondary lichen metabolites compared to solvent controls.

### Comparison of Rhythm Parameter Changes After Treatment With Lichen Secondary Metabolites

Normalized amplitude, period, phase, and dampening rate in different reporter cell models are depicted in Figures 2–4 in panels **C,D,E,F**, respectively. 2C–F shows the rhythm parameters of N44-BL cells whereas Figures 3C–F and Figures 4C–F represent the rhythm parameters of U2OS-BL and MEF-P2L cells, respectively. Irrespective of the reporter cell line, a dampening of the amplitude was observed in all cells treated with either UA or EA (Figures 2C, 3C, 4C). In all the three cell lines, significantly lowered amplitudes were observed upon treatment with 50 μg/ml of UA or EA compared to solvent controls. U2OS-BL (Figure 3C) and MEF-P2L (Figure 4C) cells treated with 10 μg/ml of UA had significantly dampened amplitudes. UA treated cells showed lower amplitudes than solvent controls and EA treated cells. An exception was observed only in the case of N44-BL cells treated with 10 μg/ml of UA and EA (Figure 2C). At 10 μg/ml EA treated cells showed lower amplitudes than UA cells treated at the same concentration. Subtle differences in period length were observed only in MEF-P2L cells treated with 10 μg/ml (shortened) of UA and 50 μg/ml of EA (lengthened; Figure 4D). EA 10 μg/ml treated MEF-P2L cells had a significantly longer period than 10 μg/ml of UA treated cells. Irrespective of the reporter cell line, in cells treated with 50 μg/ml of UA and EA significant phase differences were observed, though directional effects on phase differed between cell types (Figures 2E; Figure 3E; 4E). 50 μg/ml of UA or EA treated MEF-P2L (4E) and N44-BL (Figure 2E) cells showed significantly advanced phase compared to solvent controls whereas U2OS-BL (Figure 3E) showed significantly delayed phases upon treatment. N44-BL cells treated with 50 μg/ml of UA showed stronger phase delays than cells treated with EA at 50 μg/ml (Figure 2E). In contrast, among cells treated with 10 μg/ml of secondary metabolites, MEF-P2L cells showed significant phase advance upon treatment with 10 μg/ml of UA against solvent control (Figure 4E). Increased dampening was observed in BL reporter cells upon treatment with 50 μg/ml of UA in N44 cells and with both UA and EA in U2OS cells (Figures 2F, 3F, 4F). N44-BL cells treated with 50 μg/ml of UA had significantly higher dampening rates than EA treated cells (Figure 2F). In summary, we have found that higher concentrations of secondary metabolites of lichens had marked effects on amplitude and dampening, while period and phase showed only modest responses.

### Phase Responses of MEF *Per2:luc* Rhythms After Acute Treatment With Lichen Secondary Metabolites

**Figure 5:**
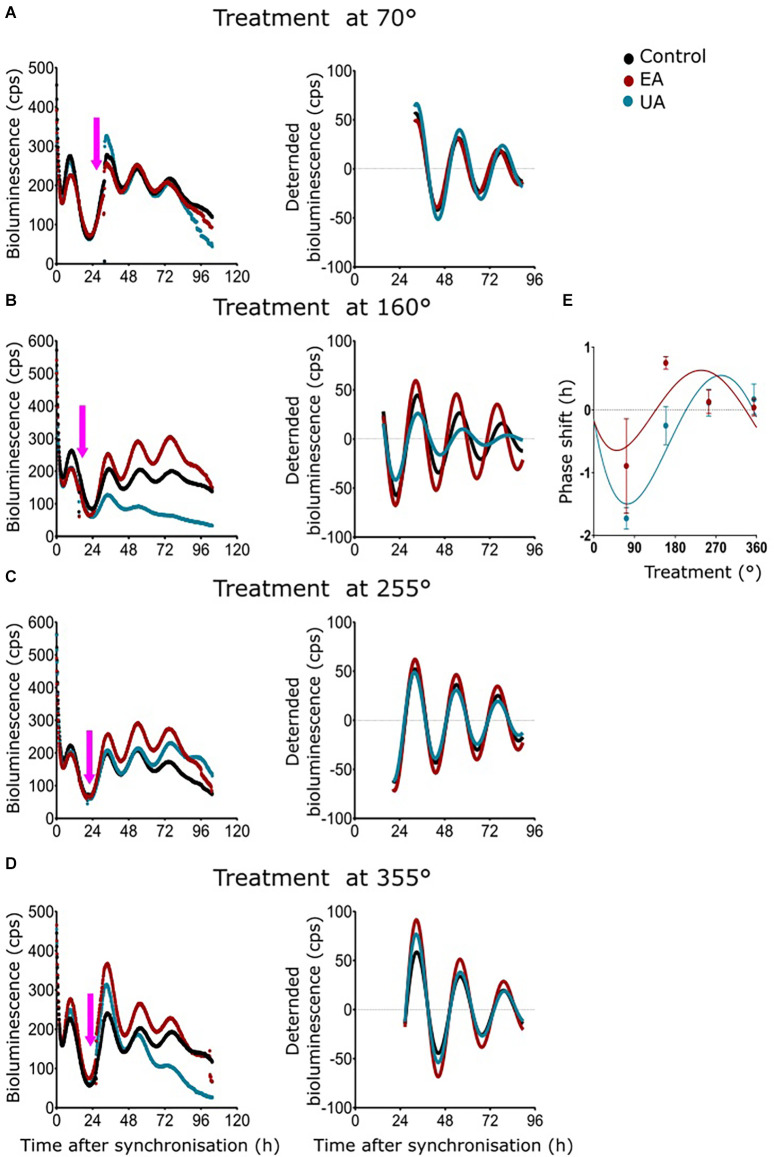
Phase response curves (PRCs) of MEF *Per2:luc* rhythms after timed treatment with secondary lichen metabolites. **(A–D)** Raw bioluminescence data (left) and normalized curve fits (right) of luminescence in MEF-P2L cells treated with 10 μg/ml of UA (blue) or EA (red) vs. solvent control (PBS/DMSO; black) at 70° **(A)**, 160° **(B)**, 255° **(C)**, or 355° **(D)** of the pre-treatment *Per2:luc* rhythm. Pink arrows depict treatment time points. **(E)** PRC of the same data set (*n* = 3 per time point; data are averages ± SEM).

A phase response curve (PRC) is the graphical illustration of phase shifts as a function of the circadian treatment phase induced by stimuli like light, food, temperature, or chemicals. As 50 μg/ml of UA and EA had profound dampening effects in all the cell models and significant differences in amplitude, period, and phase were observed from dose-dependent experiments on MEF-P2L cells treated with 10 μg/ml of UA or EA (Figure 4E), we wanted to investigate if these changes of circadian parameters are dependent on the phase of the treatment. We treated MEF-P2L cells with 10 μg/ml of UA or EA (or solvent) at different phases of the *Per2:luc* luminescence rhythm. Figure 5A shows representative data of treatments before the peak of luminescence (at ca. 70°) whereas treatment after the peak of luminescence (i.e., at ca. 160°), before the trough (at ca. 255°), and after the trough (at ca. 355°) are shown in Figures 5B–D, respectively. Panels on the left show raw data, middle panels the respective curve-fitted detrended data. Pink arrows indicate the approximate treatment times. Except the treatment at ca. 355°, PRCs reveal that UA treated cells showed advanced phase compared to EA treated cells (Figure 5E), though overall phase effects were rather small and differences in phase shifts were not significant at any time point. While PRCs on *Bmal1:luc* rhythm report that EA treated cells showed an advanced phase compared UA treated cells, the overall phase effects were inconclusive (Supplementary Figure S2). Supplementary Figure S2 depicts the representative PRC of N44-BL (Supplementary Figure S2A) and U2OS-BL (Supplementary Figure S2B) cells.

## Discussion

To test our hypothesis, we chose three different commonly used cellular circadian models, i.e., mouse embryonic fibroblasts (MEF) and hypothalamic neurons (N44) along with human osteosarcoma (U2OS) cell lines with two different circadian luciferase reporters (*Bmal1* and *Per2*). In this way, changes in circadian clock rhythms upon treatment consistently observed in all models would likely also be applicable to other tissues and cell types. Along this line, previous studies reported a broad range of pharmacological activities of UA and EA like neuroprotective, cytoprotective, and antioxidant actions in healthy neuronal, cardiac, gastric cell lines, and cytotoxic, antiproliferative and anticarcinogenic effects in different cancer cell lines (MCF-7, HeLa, HCT-116, FemX, U937, LS174, SH-SY5Y, HL-60, A2780, SK-BR-3, HT29, and U373 MG; Marante et al., 2003; de Paz et al., 2010; Bačkorová et al., 2011; Bessadottir et al., 2012; Rabelo et al., 2012; Ranković et al., 2012; Brisdelli et al., 2013; Kosanić et al., 2013; Geng et al., 2018). In a previous study, Fernández-Moriano et al. (2017) had reported the neuroprotective potential of UA and EA with strong radical scavenging properties (ORAC and DPPH tests) for the first time in 2017 in two models of central nervous system-like cells (U373-MG and SH-SY5Y cell lines). They first measured approximately 55%–60% cell viability against control after inducing oxidative stress by the exogenous H_2_O_2_. Twenty-four hours of pre-treatment with 5 μg/ml EA showed the most promising and effective cytoprotection against the oxidative damage in both of the cell models tested. Whereas, that of UA in astrocytes and neurons were 2.5 μg/ml and 1 μg/ml, respectively (Fernández-Moriano et al., 2017). In our cellular models, except U2OS cells other two cells were from mouse embryonic hypothalamic neurons and fibroblasts. Moreover, exogenous oxidative stress was not induced in any of these cells.

We observed that irrespective of the cell model and luciferase reporter type, 50 μg/ml of UA or EA significantly lowered amplitudes and accelerated the dampening of cellular circadian rhythms (Figures 2C, 3C, 4C). The lowered dampening could result from cell death by the action of higher concentrations of secondary metabolites of lichens. To find out whether lichenized substances cause cell death, we conducted a cell viability assay using AO-PI stain in the N44 and U2OS cells after 24 h pre-treatment with different concentrations of UA against PBS/ DMSO control. Our results showed that the 94.35%, 96.35%, 96.2%, 96.3%, and 71.8% (*n* = 2) N44-BL cells were viable upon 24 h pre-treatment with control along with 10 μg/ml, 25 μg/ml, 50 μg/ml, and 100 μg/ml of UA, respectively. Whereas, after 24 h pre-treatment with control along with 10 μg/ml and 50 μg/ml of UA the 98.9%, 97.8%, and 96.5% (*n* = 2) U2OS-BL cells were viable, respectively. We have observed that UA had stronger dampening effects on BL rhythms than on P2L (Figures 2A, 3A; 4A). Dose response experiments showed that BL rhythms in U2OS cells were more sensitive upon treatment with any concentration of both compounds (Figures 3A,B). At this point, the mechanism behind these effects remains unclear. Reduced amplitudes—but not stronger dampening of rhythms could result from lowered cellular levels of adenosine tri-phosphate (ATP) as ATP is required for the bioluminescence signal (Gould and Subramani, 1988). Usnic acid (10 μg/ml) treatment of the breast cancer cell line T47D and EA pre-treatment of primary neurons have been shown to reduce cellular levels of ATP by inducing the phosphorylation of adenosine monophosphate kinase (AMPK; Bessadottir et al., 2012) and proton leakage through the mitochondrial membrane (Lee et al., 2021).

Circadian rhythm alterations like disrupted sleep and dysregulated circadian gene expression patterns are early markers of neurodegenerative disease progression (Hatfield et al., 2004; Breen et al., 2014; Song et al., 2015; Musiek and Holtzman, 2016; Cronin et al., 2017; Musiek et al., 2018). Fernández-Moriano et al. (2017) reported the neuroprotective potential of UA and EA with strong radical scavenging properties in U373-MG astrocytoma and SH-SY5Y neuroblastoma cell lines. Dysfunctional astrocytes cause neurodegeneration by inducing OS, followed by downregulating *Nrf2* gene expression (Sagara et al., 1993; Fernandez-Checa et al., 1997; Itoh et al., 1997; Ishii et al., 2000; Ishii and Mann, 2014; Mann and Forman, 2015). Thus, it plays a critical role in brain health (Yamanaka et al., 2008; Macauley et al., 2011; Lian et al., 2015). Lananna et al. (2018) reported that BMAL1 protein regulates astrocyte activation *via* a cell-autonomous mechanism.

Pre-treatment of primary astrocytes with EA counteracts MPP^+^ induced COX2 (Cyclooxygenase 2) upregulation and glial activation by blocking the NF-κβ signaling pathway (Lee et al., 2021). It has been suggested that the regulation of NF-κβ activity in macrophages is negatively associated with *Bmal1* expression (Oishi et al., 2017). Besides, in U2OS cells, like CRY1, a core clock repressor, NF-κβ activation shortens the period length and represses BMAL1-mediated E-box transcription as its subunit RELA repressed the transcriptional activity of the BMAL1/CLOCK at the circadian E-box cis-element and it also altered the diurnal locomotor behavior by modifying the rhythms in the SCN (longer period; Shen et al., 2021). They have also reported that gene expression of BMAL1, IL-6, and TNF-α were upregulated by RELA. EA also imparts neuroprotective effects as its pre-treatment significantly reduces MPP^+^ induced neurite shortening in the primary neurons through reduction of ROS and reduced ATP production (Lee et al., 2021). In line with this, motor function recovery is significantly accelerated in murine Parkinson’s disease models when mice are treated with EA (Lee et al., 2021).

Usnic and evernic acids prevent ROS-induced oxidative damage in various cell types *via* activating the NRF2 signaling pathway, inhibiting caspase-3 activity, upregulating protein levels of Bcl-2, and downregulating protein level of BAX (Fernández-Moriano et al., 2015b, 2017; Krajka-Kuźniak and Baer-Dubowska, 2021). In line with their role in clock regulation, the loss of NRF2 function in mouse fibroblasts and hepatocytes alters circadian rhythms through increased Cry2 expression and repressed CLOCK/BMAL1 regulated E-box transcription in a time-dependent manner (Wible et al., 2018). Evernic acid pre-treatment restores MPP^+^ induced cell viability by inducing BCL-2 and suppressing BAX protein levels in primary murine neurons (Lee et al., 2021). In the mouse central clock, p53 acts as a transcription factor that blocks BMAL1/CLOCK binding to the Per2 promoter (Miki et al., 2013), and other mechanisms of p53-clock interaction have been described (Gotoh et al., 2014; Jiang et al., 2016). Many of these pathways offer plausible mechanisms mediating the phase alterations and changes in amplitude and dampening of circadian clock rhythms after EA/UA treatment in the tested cell models. Further experiments are necessary to test this hypothesis.

In conclusion, cellular circadian clock functions in human osteosarcoma and embryonic mouse hypothalamic neurons and fibroblasts are altered by treatment with UA and EA, suggesting that lichen secondary metabolites may provide interesting novel therapeutic options for the treatment of chronodisruption-associated diseases. Our study sets a baseline for further exploration of potential natural products for therapeutic applications of chronodisruption-associated diseases.

## Limitations

Serum shock or medium change synchronization effect against synchronization with Dex was not studied with any of the cellular models. Though we cannot exclude the interaction of the synchronization method and EA/UA treatment because of the simultaneous application.

Bioluminescence of all the dose-response and resynchronization experiments were recorded at 34°C in the plate readers, whereas bioluminescence for all the phase response experiments was recorded at 32.5°C due to the existing setup of the Lumicycle at this temperature.

## Data Availability Statement

The raw data supporting the conclusions of this article will be made available by the authors, without undue reservation.

## Author Contributions

SS: conceptualization, methodology, formal analysis, investigation, writing—original draft. CS: supervision, validation, writing—review and editing. HO: conceptualization, methodology, formal analysis, supervision, resources, software, funding acquisition, project administration, validation, visualization, writing—review and editing. MG-S and PKD: conceptualization, supervision, resources, funding acquisition, project administration, writing—review and editing. All authors contributed to the article and approved the submitted version.

## Conflict of Interest

The authors declare that the research was conducted in the absence of any commercial or financial relationships that could be construed as a potential conflict of interest.

## Publisher’s Note

All claims expressed in this article are solely those of the authors and do not necessarily represent those of their affiliated organizations, or those of the publisher, the editors and the reviewers. Any product that may be evaluated in this article, or claim that may be made by its manufacturer, is not guaranteed or endorsed by the publisher.

## Supplementary Material

Supplementary Figure 1 Lichenized secondary metabolite’s dose effects and resynchronization changes in N44-BL cells. **(A,B)** Representative raw **(A)** and representative curve fits **(B)** of *Bmal1:luc* luminescence rhythms and resynchronization effects of different concentrations of UA treatment on N44 cells. **(C,D)** Representative raw **(C)** and representative curve fits **(D)** of *Bmal1:luc* luminescence rhythms upon treatment with different concentrations of EA on N44 cells.

Supplementary Figure 2 Phase response curves (PRCs) of N44 and U2OS *Bmal1:luc* rhythms after timed treatment with secondary lichen metabolites. PRC of the N44 cells **(A)** and the U2OS cells **(B)** treated with UA (blue) or EA (red) against solvent control (PBS/ DMSO; black) at different degrees of the *Bmal1:luc* rhythm (*n* = 3 per time point; data are averages ± SEM).

## Abbreviations

EA: evernic acid
UA: usnic acid
BL: *Bmal1:luciferase*
P2L: *Per2:luciferase*
N44: hypothalamus-derived mouse N44
U2OS: human bone osteosarcoma epithelial
MEF: mouse embryonic fibroblast
Dex: dexamethasone
PRC: phase response curve.
