# Supplementary material for: Modulation of Cellular Circadian Rhythms by Secondary Metabolites of Lichens
Source: Front Cell Neurosci. 2022 Jun 23;16:907308. doi: 10.3389/fncel.2022.907308 (PMC9260025; doi:10.3389/fncel.2022.907308)
Supplement: Supplementary file 1 [file Data_Sheet_1.pdf]

SUPPLEMENTARY FIGURES

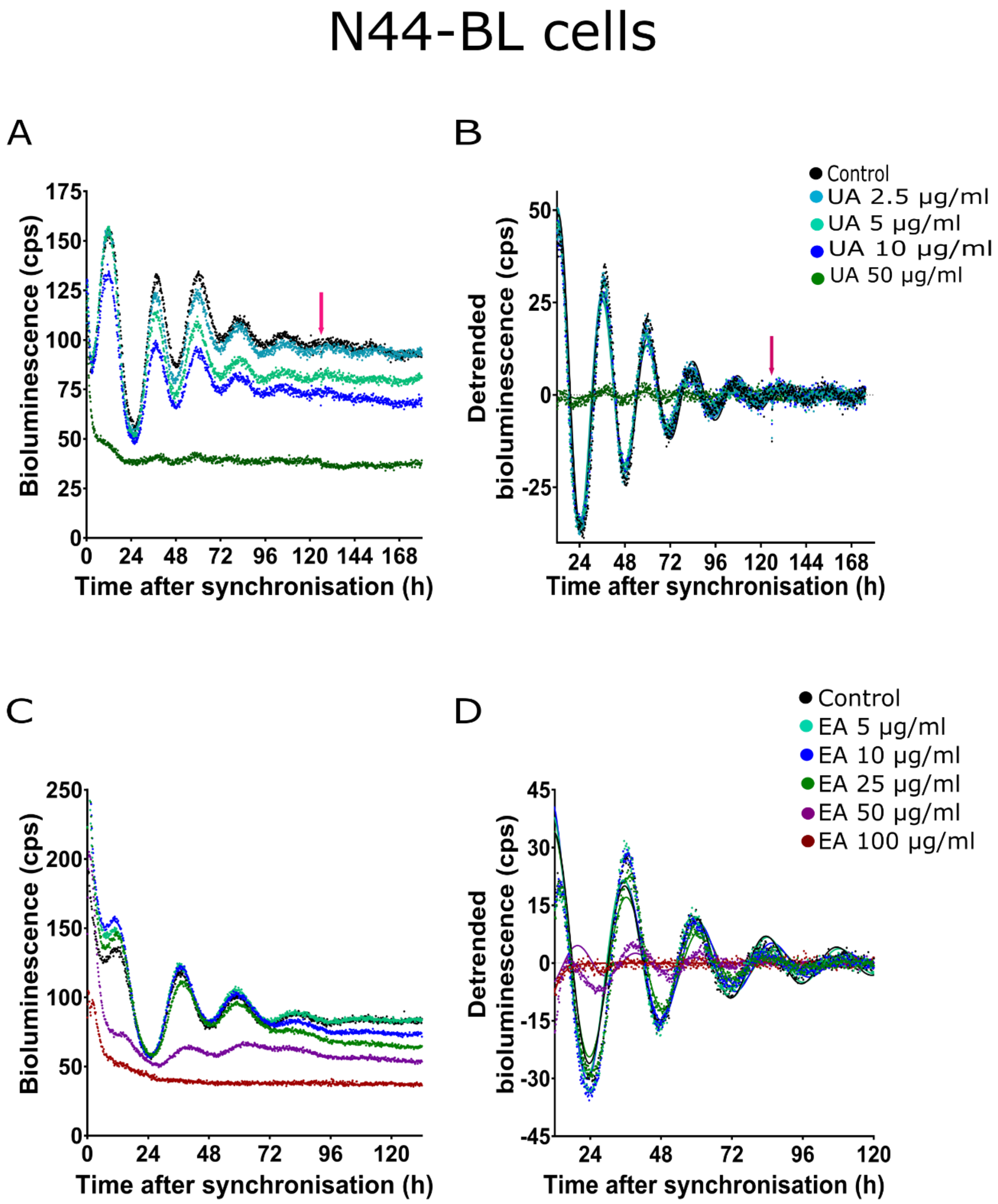

FIGURE S1 | Lichenized secondary metabolite's dose effects and resynchronization changes in N44-BL cells. (A, B) Representative raw **(A)** and representative curve fits **(B)** of *Bmal1:luc* luminescence rhythms and resynchronization effects of different concentrations of UA treatment on N44 cells. **(C, D)** Representative raw **(C)** and representative curve fits **(D)** of *Bmal1:luc* luminescence rhythms upon treatment with different concentrations of EA on N44 cells.

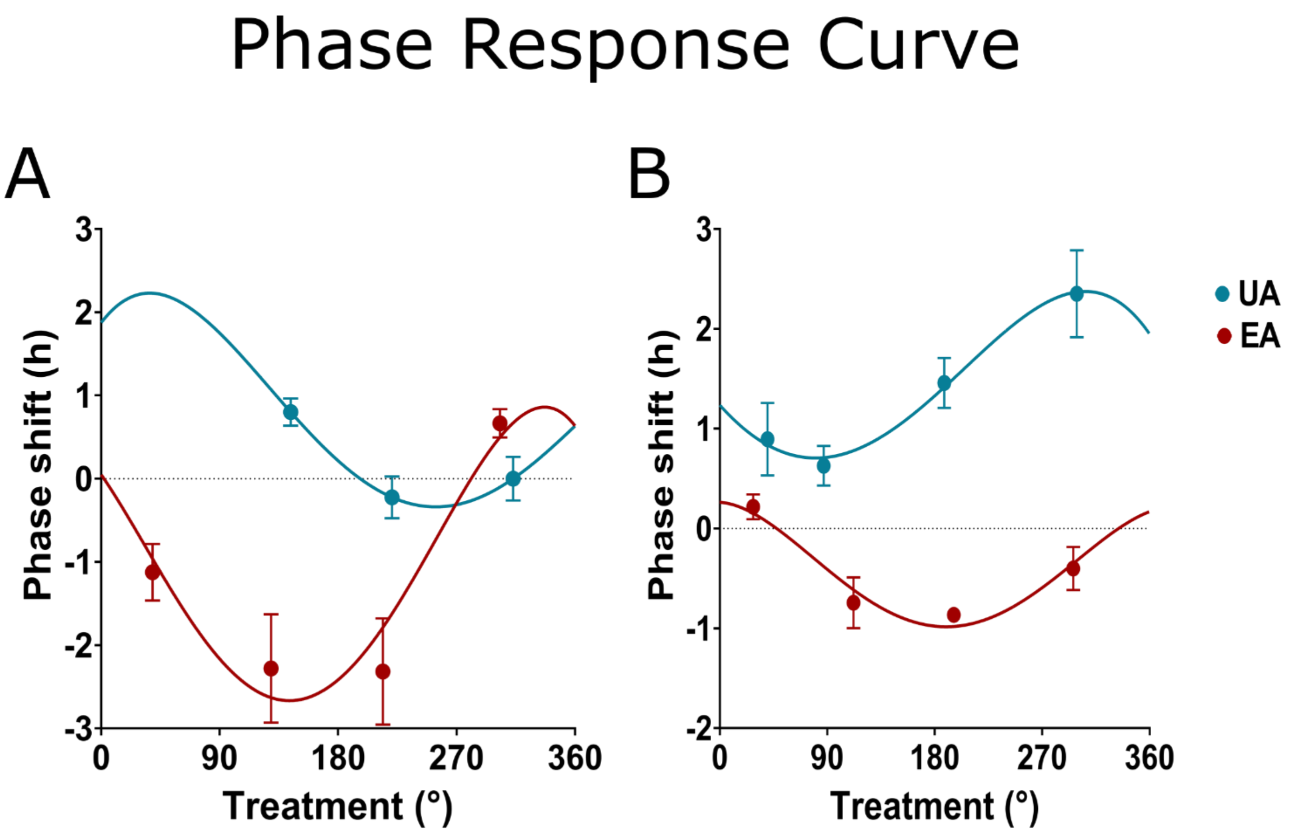

FIGURE S2 | Phase response curves (PRCs) of N44 and U2OS *Bmal1:luc* rhythms after timed treatment with secondary lichen metabolites. PRC of the N44 cells **(A)** and the U2OS cells **(B)** treated with UA (blue) or EA (red) against solvent control (PBS/ DMSO; black) at different degrees of the *Bmal1:luc* rhythm (n = 3 per time point; data are averages ± SEM).
